# Luminescent Bacteria as Bioindicators in Screening and Selection of Enzymes Detoxifying Various Mycotoxins

**DOI:** 10.3390/s24030763

**Published:** 2024-01-24

**Authors:** Elena Efremenko, Ilya Lyagin, Nikolay Stepanov, Olga Senko, Olga Maslova, Aysel Aslanli, Natalia Ugarova

**Affiliations:** Faculty of Chemistry, Lomonosov Moscow State University, Lenin Hills 1/3, Moscow 119991, Russia

**Keywords:** glucanase, molecular docking, bioluminescent bacteria, mycotoxins, detoxification

## Abstract

Interest in enzymes capable of neutralizing various mycotoxins is quite high. The methods used for the screening and selection of enzymes that catalyze the detoxification of mycotoxins should be sensitive and fast. However toxic compounds can be generated under the action of such enzymes. Thus, the assessment of the overall reduction in the toxic properties of reaction media towards bioluminescent bacteria seems to be the most reasonable control method allowing a quick search for the effective enzymatic biocatalysts. The influence of a wide range of mycotoxins and glucanases, which hydrolyze toxins with different chemical structures, on the analytical characteristics of luminescent photobacteria as a biosensing element has been studied. Different glucanases (β-glucosidase and endoglucanase) were initially selected for reactions with 10 mycotoxins based on the results of molecular docking which was performed in silico with 20 mycotoxins. Finally, the biorecognizing luminescent cells were used to estimate the residual toxicity of reaction media with mycotoxins after their interaction with enzymes. The notable non-catalytic decrease in toxicity of media containing deoxynivalenol was revealed with luminous cells for both types of tested glucanases, whereas β-glucosidase provided a significant catalytic detoxification of media with aflatoxin B_2_ and zearalenone at pH 6.0.

## 1. Introduction

Mycelial fungi are capable of causing significant damage to agricultural products [1] due to their own highly active hydrolytic enzymes and oxidoreductases. However, their ability to synthesize and secrete various mycotoxins into their growth media is particularly dangerous. Moreover, this can happen both at the stage of growth of agricultural crops and during the storage of crops after harvesting [2]. It is important that mycotoxins are preserved when using such raw materials in animal feed and further in livestock products, showing nephro-, hepato-, and neurotoxicity [3]. The variety of mycotoxins found in agricultural raw materials and products raises serious questions about the need to comply with the food biosafety of agricultural products and the development of methods for their effective detoxification [4,5].

Currently, most researchers are focused on the search for enzymes and their testing in the detoxification of mycotoxins [6]. At the same time, special attention is drawn to those enzymes that can exhibit their catalytic activity in relation to several mycotoxins at once, which are present primarily in animal feeds [7]. It is known that hydrolytic enzymes are traditionally added to animal feeds to improve the digestibility and assimilation of nutrients present in the feeds. Among such enzymes, various glucanases are most often used, and they mostly have maximum activity at pH 5.0–6.0 [8]. In this regard, it seems advisable to combine the use of these enzymes with those that can affect mycotoxins in the same pH range. It looks even more expedient to search for catalysts aimed at the detoxification of mycotoxins precisely among these digestive glucanases, the addition of which has already been approved and is being implemented in practice [9], instead of creating new recombinant enzymes acting on mycotoxins at pH 6.0 [10]. The potential possibility of such an approach to the solution of the mycotoxin problem was previously theoretically predicted for a single cellobiohydrolase based on the results of an analysis conducted in silico using the molecular docking method [11]. Recently, computer modeling of possible interaction of potential substrates (particularly mycotoxins) with enzymes became very popular [12,13,14,15]. The initial screening and selection of enzymes that have a catalytic effect on mycotoxins can be and should be carried out using computer methods [6,16]. On the one hand, this makes it possible to significantly simplify and accelerate the procedure for finding the right biocatalyst and determining its possible substrates. On the other hand, it is possible to avoid the need for contact with mycotoxins during in vitro experiments and conducting experiments that may lead to negative results (lack of catalysis) [7,15,16].

At the same time, the computer version of the search for the necessary enzymes is carried out most often by the method of molecular docking [7,15,16]. Computer models allow tracking the possibility and the energy of interaction between the active center of enzymes and mycotoxins as potential substrates.

The next stage of enzyme screening for effective effects on mycotoxins should be an express assessment of the toxicity of reaction media obtained after enzymatic catalysis [11]. At the same time, such an assessment should reflect both the residual toxicity from the mycotoxin itself and the possible toxicity generated by the resulting catalytic products [6]. It makes sense to study all the catalytic characteristics of enzymes after an obvious and confirmed decrease in the toxicity level of the reaction media [11].

The generally recognized international method of toxicity assessment is based on the use of choline esterase inhibition [17] and photobacterium cells, the level of bioluminescence intensity of which depends on the type and concentration of the toxin appearing in their microenvironment [18]. Generally, both methods give a quick response to the presence of toxic compounds. In our previous comparative investigation of choline esterase and immobilized luminous bacteria *Photobacterium phosphoreum* [19] in the detection of four mycotoxins, it was established that biosensors on the basis of photobacterial cells had better analytical characteristics. Moreover, it was shown that the cells can be applied for the estimation of the resulting toxicity of the medium obtained after the hydrolytic reaction of hexahistidine-tagged organophosphorus hydrolase with zearalenone.

**Figure 1:**
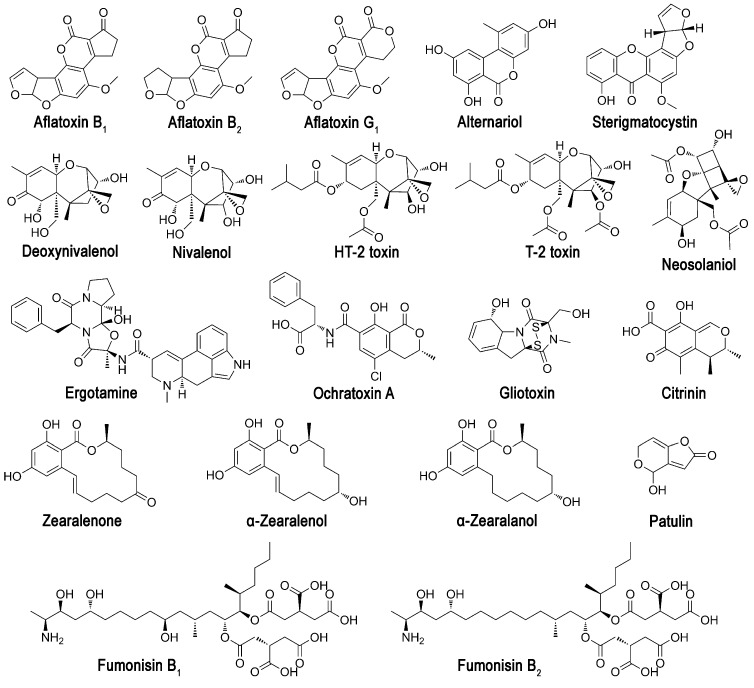
Chemical structures of mycotoxins considered in the work.

This study was aimed at confirming the possibility of using the same immobilized luminous bacteria *P. phosphoreum* to assess the toxicity of mycotoxins, the number of which was increased to 10. In the presence of a response to mycotoxin toxicity in bioluminescent cells, these biosensitive elements were involved in the wet screening of potential substrates for enzymes. The range of possible substrates for the same enzymes was evaluated by molecular docking in the analysis of 20 mycotoxins (Figure 1), and two different glucanases were considered as enzymes capable of catalyzing the detoxification of mycotoxins.

## 2. Materials and Methods

### 2.1. Materials

Ten mycotoxins (Figure 1) were involved in wet experiments with enzymes and luminous cells: AFB1 and AFB2—aflatoxins B_1_ and B_2_; αZAL—α-zearalanol; CIT—citrinin; NIV—nivalenol; DON—deoxynivalenol; SCN—sterigmatocystin; OCH—ochratoxin A; ZEA—zearalenone; PAT—patulin. Solutions of AFB1, AFB2, DON, SCN (Sigma-Aldrich, Darmstadt, Germany); NIV, OCH (Cayman Chemical, Ann Arbor, MI, USA); αZAL, CIT, PAT, and ZEA (Fermentek, Jerusalem, Israel) in methanol were prepared freshly before experiments. A solution of β-glucosidase (10 kU/mL) from *Myceliophtora fergusii* was supplied by BioPreparat (Voronezh, Russia) and used as received without additional purification. Purified endoglucanase (5 mg/mL) from *Trichoderma atroviride* was provided by Dr. Margarita Semenova (Laboratory of Prof. Arkady P. Sinitsyn, Moscow State University, Moscow, Russia).

Inorganic salts and glycerol (Chimmed, Moscow, Russia), peptone and yeast extract Difco (Becton, Dickinson and Company, Franklin Lakes, NJ, USA), and poly(vinyl alcohol) 16/1 with M.w. 84 kDa (Sinopec Corp, Beijing, China) were purchased from the mentioned companies. *Photobacterium phosphoreum* B-1717 was obtained from the All-Russian Collection of Industrial Microorganisms (https://vkpm.genetika.ru/, accessed on 19 January 2024).

### 2.2. Production of Immobilized Photobacteria

The biomass of *P. phosphoreum* cells was accumulated at 18 °C for 22 h under aerobic and constant agitation conditions (60 rpm, IRC-1-U temperature-controlled shaker, Adolf Kuhner AG Apparatebau, Basel, Switzerland) as described previously [19]. The Farghaly growth medium recommended for the photobacterial cells was used for this purpose. The cells grown up to an optical density of 0.75 at the wavelength of 660 nm were separated from the culture medium by centrifugation (5000 rpm, 15 min, J2-21 centrifuge, Beckman, Brea, CA, USA). This step was followed by the immobilization of the bacterial biomass into poly(vinyl alcohol) (PVA) cryogel according to a previously developed procedure [19]. Briefly, cells were thoroughly mixed with a 10% (*w*/*v*) aqueous PVA solution to obtain a 1% (*w*/*w*) concentration of bacterial cells, frozen at –70 °C for 24 h in 96-well microplates (0.2 mL/well) and thawed at +4 °C before use.

### 2.3. Analysis of Toxicity by Luminescence Measurements

The toxicity of stock solutions of mycotoxins as well as reaction media after enzymatic treatment was determined using a previously published protocol [19] with minor modifications. Briefly, a weighted piece of a granule with immobilized *P. phosphoreum* cells was introduced into 0.1 mL of a 2% NaCl solution containing 0–1000 μg/mL of mycotoxin (0.5–5 mg/mL in methanol) or into an equal volume of mycotoxin reaction media. The residual intensity of bioluminescence (*I*/*I*_0_) was measured after 0.5 h of exposure with a 3560 microluminometer (New Horizons Diagnostics Co., Columbia, MD, USA) and expressed as a percentage of the baseline signal (*I*_0_). For a control, the same procedures were performed with corresponding concentrations of methanol in a 2% NaCl solution or with reaction media without mycotoxins; the obtained values of residual intensity were used for the normalization of other means into a single unified scale. The results of three independent experiments were averaged and presented as means ± standard deviation (SD). The influence of residual concentrations of methanol used for the preparation of stock mycotoxin solutions was specially estimated and taken into account.

### 2.4. Treatment of Mycotoxins with Enzymes

Enzyme reactions generally followed the protocol published previously [11] with minor modifications. Briefly, 12 μL of mycotoxin in methanol (5 mg/mL AFB1, AFB2, CIT, αZAL, NIV, PAT, ZEA; 2 mg/mL OCH; 1 mg/mL DON; or 0.5 mg/mL SCN) was mixed with 96 μL of 0.1 M phosphate buffer (pH 6). After that, 12 μL of β-glucosidase (10 kU/mL) or endoglucanase (5 mg/mL) was added. The reaction vessels were maintained at +25 °C for 0.5 h with constant stirring (800 rpm). Then, a 0.1 mL aliquot was sampled for toxicity measurement. For a control, the same reaction media were inoculated with 12 μL of the same buffer instead of enzymes and treated similarly.

### 2.5. Computer Modeling of Mycotoxin–Enzyme Interaction

Simulations were performed using a previously published method [11]. Briefly, organic compounds drawn in ChemBioDraw and minimized in ChemBio3D with the MM2 force field (ver. 12.0, CambridgeSoft, Cambridgeshire, UK) were converted from the PDB format to the PDBQT format using AutoDockTools (as part of MGLTools ver. 1.5.6, available at https://ccsb.scripps.edu/mgltools/, accessed on 19 January 2024) [20]. Overall, 20 mycotoxins (aflatoxin B_1_, AFB1; aflatoxin B_2_, AFB2; aflatoxin G_1_, AFG1; alternariol, ALT; α-zearalanol, αZAL; α-zearalenol, αZOL; citrinin, CIT; deoxynivalenol, DON; ergotamine, ErgT; fumonisin B_1_, FMB1; fumonisin B_2_, FMB2; gliotoxin, GLT; HT2 toxin; neosolaniol, NEO; nivalenol, NIV; ochratoxin A, OCH; patulin, PAT; sterigmatocystin, SCN; T2 toxin; zearalenone, ZEA) and common glucanase substrate (pNP-cellobiose) were generated.

The known crystallographic structure of glucohydrolase from *Trichoderma reesei* (PDB 4I8D) was obtained from the Protein Data Bank. The amino acid sequence of glucanase from *Thermothelomyces thermophilus* (former *Myceliophthora thermophila*) was obtained from UniProt (H2B658), and its structure was predicted using the I-TASSER server (ver. 5.1, available at https://zhanggroup.org/I-TASSER/, accessed on 19 January 2024) [21]. The proper atom charge being relevant to simulated reaction conditions (pH 6) was assigned using the PDB2PQR (ver. 3.6.1) and Adaptive Poisson-Boltzmann Solver (APBS, ver. 3.4.1) servers (available at https://server.poissonboltzmann.org/pdb2pqr/, accessed on 19 January 2024) [22,23] with the PARSE force field and default settings. After that, structures were converted from the PQR format to the PDBQT format using AutoDockTools.

Molecular docking of the mycotoxins to the selected enzymes was performed using AutoDock Vina (ver. 1.1.2, available at http://vina.scripps.edu/, accessed on 19 January 2024) [24] with default program options on a desktop computer equipped with an Intel Pentium Dual-Core CPU E5400 2.7 GHz and 3 GB of available memory. The grid box was approximately centered on the enzyme center of mass and occupied up to 400 nm^3^ (7 × 7 × 7 nm). By default, 12 models with the lowest affinities (i.e., binding energies) were chosen for each ligand–enzyme pair. Distances between atoms of catalytic groups in the active center of the enzyme and “labile” atoms of ligands were measured and visualized with PyMOL Molecular Graphics System (ver. 1.7.6, Schrödinger, LLC, New York, NY, USA). Statistical analysis was realized and illustrated in OriginPro (v.9.4.2.380, OriginLab, Northampton, MA, USA).

The GAMESS-US package (ver. 2017 R1, available at https://www.msg.chem.iastate.edu/GAMESS/, accessed on 19 January 2024) [25] was used for quantum mechanical calculations of molecular surfaces of different mycotoxins as described previously [6] (i.e., unrestricted Hartree–Fock, B3LYP hybrid functional, basis 6-31G*, single *p*-type polarization of hydrogens and diffusion of their *s* shell, Pipek–Mezey population localization of orbitals). Preliminary hydrogen atoms, which were omitted in the preceding stages, were added to the selected mycotoxin poses automatically by PyMOL taking into account proper valence and controlled manually. Calculations were performed using Supercomputer “Lomonosov-2” of Lomonosov Moscow State University [26], utilizing up to 4 Intel Haswell-EP E5-2697v3 2.6 GHz CPUs with Intel MPI Library (ver. 2019.9, Intel, Santa Clara, CA, USA) and 4 NVidia Tesla K40M GPUs with Cuda (ver. 8.0, Nvidia, Santa Clara, CA, USA).

## 3. Results

### 3.1. The Response of Bioluminescent Cells to the Presence of Different Mycotoxins

**Figure 2:**
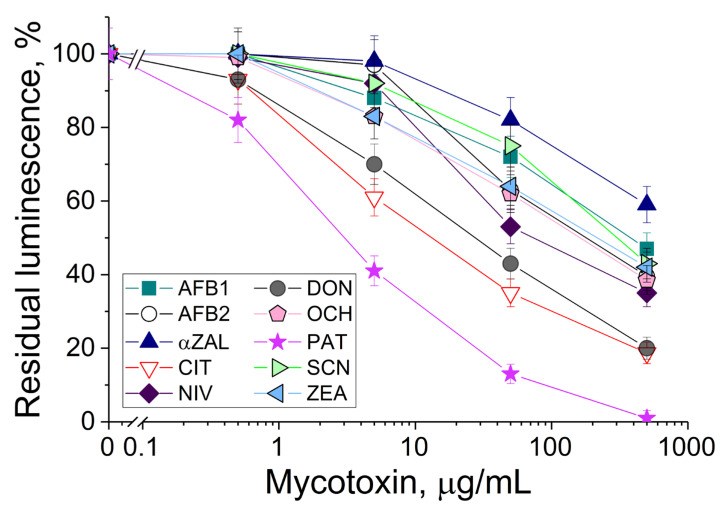
Residual intensity of bioluminescence of immobilized *P. phosphoreum* cells in the presence of various mycotoxins: AFB1 and AFB2—aflatoxins B_1_ and B_2_; αZAL—α-zearalanol; CIT—citrinin; NIV—nivalenol; DON—deoxynivalenol; SCN—sterigmatocystin; OCH—ochratoxin A; ZEA—zearalenone; PAT—patulin.

The *P. phosphoreum* cells immobilized in PVA cryogel were exposed to solutions with different concentrations of tested mycotoxins under the same conditions (Figure 2). The number of mycotoxins as compared to the previous investigations [19,27] was increased two-fold. The bioluminescence decreased when concentrations of mycotoxins were increased. The most frequent linear ranges of detection of various mycotoxins were within 0.5–500 μg/mL.

The decrease in bioluminescence in the presence of all aflatoxins was insignificant, although these mycotoxins are considered the most toxic compounds for humans and animals due to their carcinogenicity. Interestingly, patulin demonstrated the highest toxicity for the luminous cells, and this result positively correlates with the data known for suspended luminous cells *Vibrio qinghaiensis* [27]. A similar tendency in the strong decrease in the bioluminescence of *P. phosphoreum* (Figure 2) and *V. qinghaiensis* cells [27] was revealed for ochratoxin A and citrinin.

The notable negative action of deoxynivalenol on the *P. phosphoreum* cells significantly differed from the results published for other luminescent cells, where the bioluminescence inhibition of *V. qinghaiensis* cells was at a middle level [27]. Therefore, that is quite a valuable result from the practical point of view, because the development of discriminative analysis of mycotoxin(s) is made possible by combining different reactions of luminous cells with various individual mycotoxins and implementing chemometrics.

### 3.2. In silico Investigation of Interactions between Mycotoxins and Enzymes

**Figure 3:**
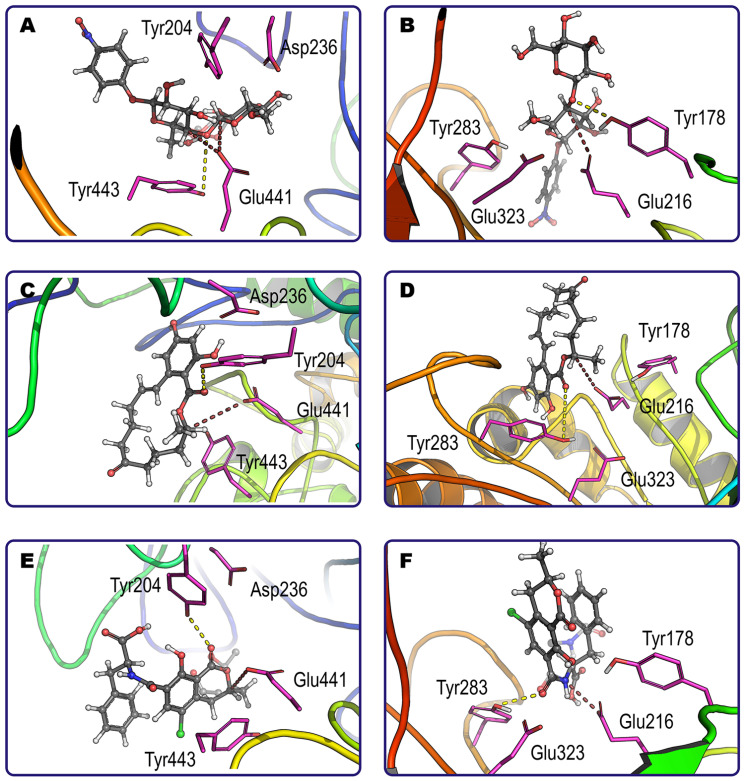
Examples of productive binding of pNP-cellobiose (**A**,**B**), zearalenone (**C**,**D**) and ochratoxin A (**E**,**F**) within the active center of glucanase 4I8D (**A**,**C**,**E**) and glucanase H2B658 (**B**,**D**,**F**) at pH 6. Catalytically essential tetrads of amino acids are shown by magenta sticks, while corresponding shortest distances and possible directions for nucleophilic attack and proton donating are indicated by dashed lines of brown and yellow color, respectively. The main substrate binding pose is solidly colored (carbon atoms are grey), and alternative model locations (only for the labile group for simplification) are transparent.

**Figure 4:**
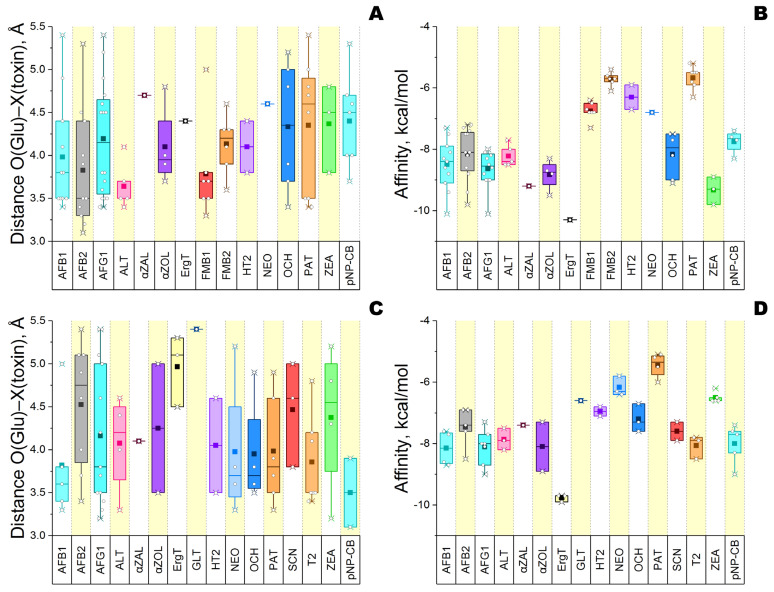
Qualitative analysis of different mycotoxins binding to the active center of glucanase 4I8D (**A**,**B**) and glucanase H2B658 (**C**,**D**) at pH 6. A common substrate (pNP-cellobiose) was used as a reference. Designations: the interquartile ranges (25–75%) are enclosed by rectangles, which are divided by a line corresponding to the median value; ■—the mean value over all datapoints; ○—the value for the individual model. Each color corresponds to a certain mycotoxin present on the abscissa axis: AFB1, AFB2, and AFG1—aflatoxins B_1_, B_2_, and G_1_; ALT—alternariol; αZAL—α-zearalanol; αZOL—α-zearalenol; ErgT—ergotamine; FMB1—fumonisin B_1_; FMB2—fumonisin B_2_; GLT—gliotoxin; HT2 toxin; NEO—neosolaniol; OCH—ochratoxin A; PAT—patulin; SCN—sterigmatocystin; T2 toxin; ZEA—zearalenone; pNP-CB—pNP-cellobiose.

Computer modeling of interactions between 20 mycotoxins and glucanases 4I8D and H2B658 was performed. The effective binding of most mycotoxins (14 and 15, respectively) as well as regular glucanase substrate pNP-cellobiose was demonstrated (Figure 3 and Figure 4). It was established that both enzymes cannot form productive complexes with three common mycotoxins, namely citrinin, deoxynivalenol, and nivalenol.

It is also reasonable to discard cases of a single binding pose since they have a higher probability of false-positive results. In this way, a total of 11 and 13 “enzyme/mycotoxin” complexes could be considered as productive for glucanase 4I8D and H2B658, respectively. These two groups have nine common mycotoxins (three aflatoxins, alternariol, α-zearalenol, HT2, ochratoxin A, patulin, and zearalenone) for which the enzymes have a close specificity according to estimates of binding energy, excepting zearalenone which binds more effectively with glucanase 4I8D than with H2B658. 

For both investigated enzymes, the lowest energy of interaction (affinity) reflecting the best level of enzymatic specificity was revealed towards ergotamine, and the highest energy (i.e., the worst specificity) was revealed towards patulin.

Interestingly, although both enzymes have a similar range of substrates, they sometimes exhibit different individual preferences among mycotoxins with several labile sites. For example, zearalenone has a single point of possible modification by both glucanases, namely its lactone ring (Figure 3C,D). Contrarily, ochratoxin A has two sites (amide bond and lactone ring) that are preferred by glucanases H2B658 and 4I8D, respectively (Figure 3E,F). As a result, the end-products that appear in mycotoxin hydrolysis depend on the enzyme applied and may reveal different toxicity profiles. Other such examples were ergotamine, HT2, neosolaniol, patulin, and pNP-cellobiose. Enzyme specificity is likely to be a result of the superimposition of multiple factors having cumulative and/or synergetic action: the geometry of the molecule and active site (i.e., steric hindrances); interactions of other atoms of the mycotoxin with side groups of the enzyme active site (i.e., Van der Waals, ionic, and hydrogen bonding); other factors.

In addition, the enzymes used as models differed in the amino acids involved in catalysis, among which Tyr/Glu/Asp/Tyr and Tyr/Glu/Glu/Tyr in glucanase 4I8D and H2B658, respectively, were the most important. The different amino acids are arranged in such a way that they can participate in simultaneous interaction with the labile group of a number of substrates. For example, aflatoxin G_1_ and alternariol can be affected by both “centers” in both enzymes. At the same time, both “centers” were functional with aflatoxin B_2_, HT2, neosolaniol, ochratoxin A, patulin, sterigmatocystin, and T2 only in glucanase H2B658. Thus, the efficiency of the enzyme as well as a number of end-products can vary to a certain degree depending on the specific action of glucanase used for mycotoxin hydrolysis.

### 3.3. Bioluminescent Sensing of Results of Enzymatic Action on Mycotoxins

**Figure 5:**
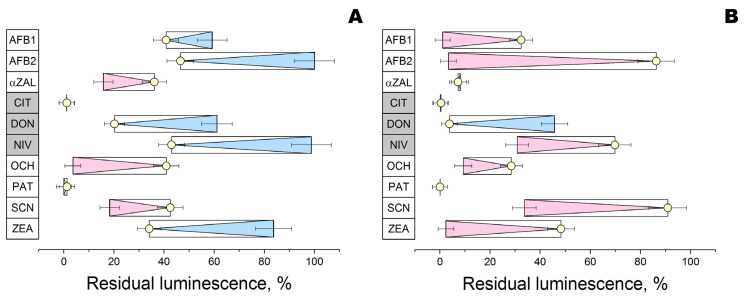
Effect of reaction medium of β-glucosidase (**A**) or endoglucanase (**B**) with different mycotoxins (AFB1 and AFB2—aflatoxins B_1_ and B_2_; αZAL—α-zearalanol; CIT—citrinin; DON—deoxynivalenol; NIV—nivalenol; OCH—ochratoxin A; PAT—patulin; SCN—sterigmatocystin; ZEA—zearalenone) on luminescence of *P. phosphoreum*. Control reaction media with mycotoxins (**○**) were exposed under the same conditions but without the addition of enzymes. The bioluminescence intensity of cells similarly exposed to methanol (or methanol with enzyme) in concentrations relevant to the reaction medium was assumed as 100%. Increase (blue) and decrease (pink) in residual luminescence as compared to control are highlighted by triangles to the right and left, respectively. Mycotoxins that are not substrates according to computer modeling are placed in grey boxes.

To test computer modeling results, two glucanases were introduced into a reaction with 10 mycotoxins at pH 6 followed by an analysis of residual toxicity on photobacterial cells possessing bioluminescence (Figure 5). From the standpoint of toxicity to the photobacterial cells, the enzymatic action of the two tested glucanases was directed oppositely in most cases (5 of 10 mycotoxins).

Based on the residual bioluminescence level, both enzymes had no effect on citrinin (a “non-substrate” according to computer modeling) and patulin (“possible substrate”). In addition, both enzymes decreased the toxicity of deoxynivalenol (“non-substrate”), while structurally relevant nivalenol (“non-substrate” having additional OH-group in its structure) was detoxified by one enzyme and stimulated in its toxicity by the other glucanase. Among other mycotoxins affected in opposite directions by the two enzymes, aflatoxins (B_1_ and B_2_) and zearalenone (as well as α-zearalanol) should be mentioned. Interaction with both glucanases increased the toxicity of reaction media with ochratoxin A and sterigmatocystin.

## 4. Discussion

Luminous cells are actively used to assess the toxicity of agricultural products contaminated with mycotoxins [27,28]. However, it was shown for the first time in this work that immobilized photobacteria can be used to determine the level of toxicity of media with a higher number (in our case, ten) of different mycotoxins (Figure 2). In fact, the versatility of this biosensitive element for detecting the presence of a wide range of toxins synthesized by fungi of different genera, which can contaminate various samples of agricultural raw materials and products, was demonstrated. The obtained dependencies of the residual bioluminescence of photobacterial cells also allow us to compare the toxicities of different mycotoxins with each other in relation to the biosensitive element used.

It is interesting to note that the toxic action of mycotoxins and effects revealed in correspondence to their concentrations on immobilized photobacterial cells of *P. phosphoreum* differed from those that are considered to be toxic for people [6,29] and were published for other luminous bacteria (*Vibrio qinghaiensis* [27]). So, the toxicity level depends on the test object used for its estimation and the object’s sensitivity to certain toxic compounds. The knowledge about “preferences” in the sensing of various luminous bacteria as biorecognizing elements can be very useful for the development of discriminative biosensors applicable for the accurate identification of individual mycotoxins in complicated media. Discriminative biosensing is well established for a number of compounds [30].

Enzymatic modification of mycotoxins may result in a loss of their toxicity [11] due to decreased interaction of modified products with intracellular target(s). Otherwise, such modification can stimulate toxic action, e.g., as in the known case of cytochromes [6]. Thus, bioluminescent bacterial analysis of the toxic effects of end-products after the enzymatic treatment of mycotoxins can be considered as a good laboratory practice.

Computer modeling allows only a preliminary selection of possible reactive partners [11], thus improving the efficiency of the following procedures due to the exclusion of less reactive or inconvenient “enzyme/mycotoxin” pairs. All amino acid sequences of enzyme molecules selected for modeling were substantially different (identity was less than 5% for all pairs). Theoretically, that could provide some minimal basis for estimating applicability and more importantly the possible limitations of computer modeling for such enzyme systems. The newly selected glucanases 4I8D and H2B658 were found to be much more active as compared to glucanase 1RQ5 published previously [11]. The common mycotoxins hydrolyzable by these three enzymes at pH 6 were ochratoxin A, patulin, and zearalenone (plus aflatoxin B_1_ at pH 7.5), while gliotoxin and sterigmatocystin were common only for 1RQ5 and H2B658. The cross-reactivity of enzymes from this work and [11] seems to be narrower. However, it is rather apparent since the mycotoxin pool was enlarged almost two-fold in the current work (20 vs. 11). Noteworthily, the affinities of glucanase 1RQ5 for binding ochratoxin A, sterigmatocystin, and zearalenone were orders of magnitude better compared to those of both 4I8D and H2B658.

Interestingly, both citrinin and deoxynivalenol did not form productive enzyme–substrate complexes with glucanase 1RQ5 also [11]. However, according to the current work, citrinin was not modified by both glucanases, while deoxynivalenol was not transformed but rather bonded by proteinous molecules, thus decreasing its bioavailability/biotoxicity. Nivalenol, being a close structural analog of deoxynivalenol, was more dependent on the chemical structure of the enzyme with which it entered into non-catalytic interaction. In this regard, only in the presence of β-glucosidase was a decrease in the toxicity of the medium with this mycotoxin observed.

In addition, patulin is worth mentioning since it was revealed as a substrate in the current and previous work [11], but no enzymatic activity was observed with glucanases in the current work. According to its affinity for all three glucanases, it is a poor substrate with a binding constant in a range of mmol/L. It seems that the observable lack of its conversion was caused by low specific enzyme activity.

Unexpectedly, the catalytic conversion of ochratoxin A and sterigmatocystin by both glucanases increased their toxicity for bioluminescent bacteria cells (Figure 5). This result can be of great practical importance since many agricultural products are treated with glucanases, which thereby could stimulate the toxicity of contaminated sources, for example, animal feeds.

Even more unobvious situations can take place for other mycotoxins like aflatoxins as well as zearalenone and its derivatives (Figure 5). There may be several possible mechanisms and combinations thereof: (1) multiple points within a mycotoxin structure for enzymatic modification that can occur simultaneously and/or competitively (aflatoxins are more likely to act in this pathway); (2) preferable binding of a modified product (or even unmodified substrate) to one enzyme as compared to the other one; (3) differing pattern of cellular transport of two enzymes and/or their complexes with mycotoxins/products. To solve this ambiguity, the structure of products, their toxicity profile, and their molecular targets within cells (at least, such targets for mycotoxins themselves) should be addressed. In any case, toxicity sensing with bioluminescent bacterial cells should be required for all products possibly contaminated with mycotoxins.

## 5. Conclusions

As a result of this study, it was found that *P. phosphoreum* cells immobilized in PVA cryogel can be effectively used to estimate the presence of ten mycotoxins in a wide enough range of their concentrations (from tenths to hundreds of micrograms per liter) and the efficiency of detoxification of several mycotoxins by the tested glucanases. It was established that contact of both tested glucanases (β-glucosidase and endoglucanase) with deoxynivalenol can result in a decrease in medium toxicity due to non-specific binding. Moreover, β-glucosidase can provide a significant catalytic detoxification of media with aflatoxins and zearalenone at pH 6. The estimations of mycotoxins’ presence and levels of enzymatic detoxification can be performed rapidly and accurately. This work confirms the high potential of immobilized bioluminescent cells in investigations of mycotoxin toxicity and new enzymes capable of its elimination. The obtained results are of practical significance because complexes of various glucanases including both endoglucanases and β-glucosidases are widely used as feed additives for many animals, where the presence of mycotoxins is under special control.

## Data Availability

Data are contained within the article.
